# Coexistence of Polyarteritis Nodosa of the Vulva and Retina in a Behçet's Disease Patient: A Case Report

**DOI:** 10.7759/cureus.16096

**Published:** 2021-07-01

**Authors:** Amal O Al-Balbeesi, Rama A Alhallaf, Najlaa A Alsubeeh, Amany A Fathaddin, Asma A Bedaiwi, Mohammed A Omair

**Affiliations:** 1 Dermatology, King Khalid University Hospital, Riyadh, SAU; 2 General Practice, King Saud University, Riyadh, SAU; 3 Dermatopathology, King Khalid University Hospital, Riyadh, SAU; 4 Rheumatology, King Khalid University Hospital, Riyadh, SAU

**Keywords:** ocular manifestations, female genitalia, ulcer, behcet disease, polyarteritis nodosa

## Abstract

Polyarteritis nodosa (PAN) is a multisystem disease that may affect the vessels of multiple organ systems. It has clinical variants including single-organ disease and cutaneous-only PAN. To our knowledge, this is a unique case report describing the coexistence of PAN of the vulva and retina in a Behçet's disease (BD) patient.

We report a case of a 31-year-old Lebanese woman with painful genital ulcers and multiple oral aphthae associated with arthralgia, light flashes, blurry vision, and photosensitivity. There were well-defined, punched-out erosions over the buccal and gingival mucosa; specifically, multiple punched-out, deep ulcers with unremarkable borders and black eschar involving two-thirds of both labia majora and minora sparing the clitoris with bilateral inguinal lymphadenopathy. Dilated fundus examination showed a few cotton wool spots and intraretinal hemorrhage. Fundus fluorescein angiography showed multiple arteriolar infarctions involving the macula in both eyes, more so in the right eye. Vulvar biopsy was consistent with PAN due to the involvement of a medium-sized subcutaneous artery and showed neutrophilic infiltration of its wall. Stain for elastic lamina showed medium-sized subcutaneous artery involvement. After ruling out infectious aetiologies, she was managed by intravenous pulse methylprednisolone 1,000 mg for three days, followed by oral prednisolone 50 mg with a slow taper, oral colchicine 0.5 mg twice daily, and adalimumab 40 mg once every two weeks to stop the progression of the ocular insult and genital mutilation. There was significant improvement of the ulcer with no new cutaneous or systemic manifestations.

This case report highlights the importance of considering PAN-like lesions in cases of Behçet's disease. We emphasized the addition of cutaneous PAN as one of the cutaneous manifestations of BD.

## Introduction

Polyarteritis nodosa (PAN), first described in 1866 by Küssmaul and Maier, is a multi-system necrotizing vasculitis predominantly affecting medium‐sized arteries [1]. It has clinical variants including single-organ disease and cutaneous-only PAN. Moreover, its symptomatology varies widely. The most common findings are general symptoms in the form of fatigue, fever, malaise, weight loss, and arthralgias in 93.1% of the patients, neurologic manifestations such as peripheral neuropathy in 79.0% of patients, and skin lesions in 49.7% of the patients. Virtually any system, including the eyes, can be affected [2]. A handful of cases in the literature have reported female genital tract involvement, mainly affecting the cervix and uterus [3]. In 1998, Buekers et al. reported the first case of PAN with vaginal ulceration as a single system disease [4].

Behçet’s disease (BD) is a chronic, relapsing, autoimmune inflammatory disorder that involves different organ systems [5]. It can be difficult and challenging to diagnose BD because there are no single laboratory tests or pathognomonic findings to aid diagnosis [6]. The International Criteria for BD, known as the ICBD criteria, is the classification/diagnosis criteria of choice to help the diagnosis of BD [5]. According to the criteria, a patient must get 4 points from the following list of items to be classified as having BD: oral aphthosis, genital aphthosis, and ocular lesions are each assigned 2 points, while cutaneous manifestations, vascular manifestations, neurological manifestations, and positive pathergy test are each assigned 1 point [5,7]. It is important to keep in mind that even if a patient is classified by the criteria set, this does not mean that the individual necessarily has BD [5].

The most common ocular presentation in BD is uveitis. It involves the anterior and/or the posterior segment of the eye. Anterior uveitis (AU), also known as iridocyclitis, is the most common ocular manifestation in BD. AU is rarely isolated and is often accompanied by posterior segment involvement [6]. Posterior segment findings may include the presence of vitritis (hyalitis), macular edema in the retina caused by vascular leakage, foci of necrotizing retinitis, and retinal vasculitis (RV). RV is mainly venous and often occlusive, which is pathognomonic for BD [6]. The cutaneous manifestations of BD may have many forms including inflammatory pustules, pseudo-folliculitis, acneiform rash, erythema nodosum, pyoderma gangrenosum-like lesions, Sweet-syndrome-like lesions, superficial migratory thrombophlebitis, palpable purpuric lesions of necrotizing vasculitis, and suppurative panniculitis [7-9]. In the literature, there are few reports demonstrating the coexistence of cutaneous polyarteritis-nodosa in Behçet's disease patients [8-11]. Here, we present a unique case of the coexistence of BD and cutaneous PAN affecting the genitalia in the form of labial ulcers with retinal involvement.

## Case presentation

**Figure 1:**
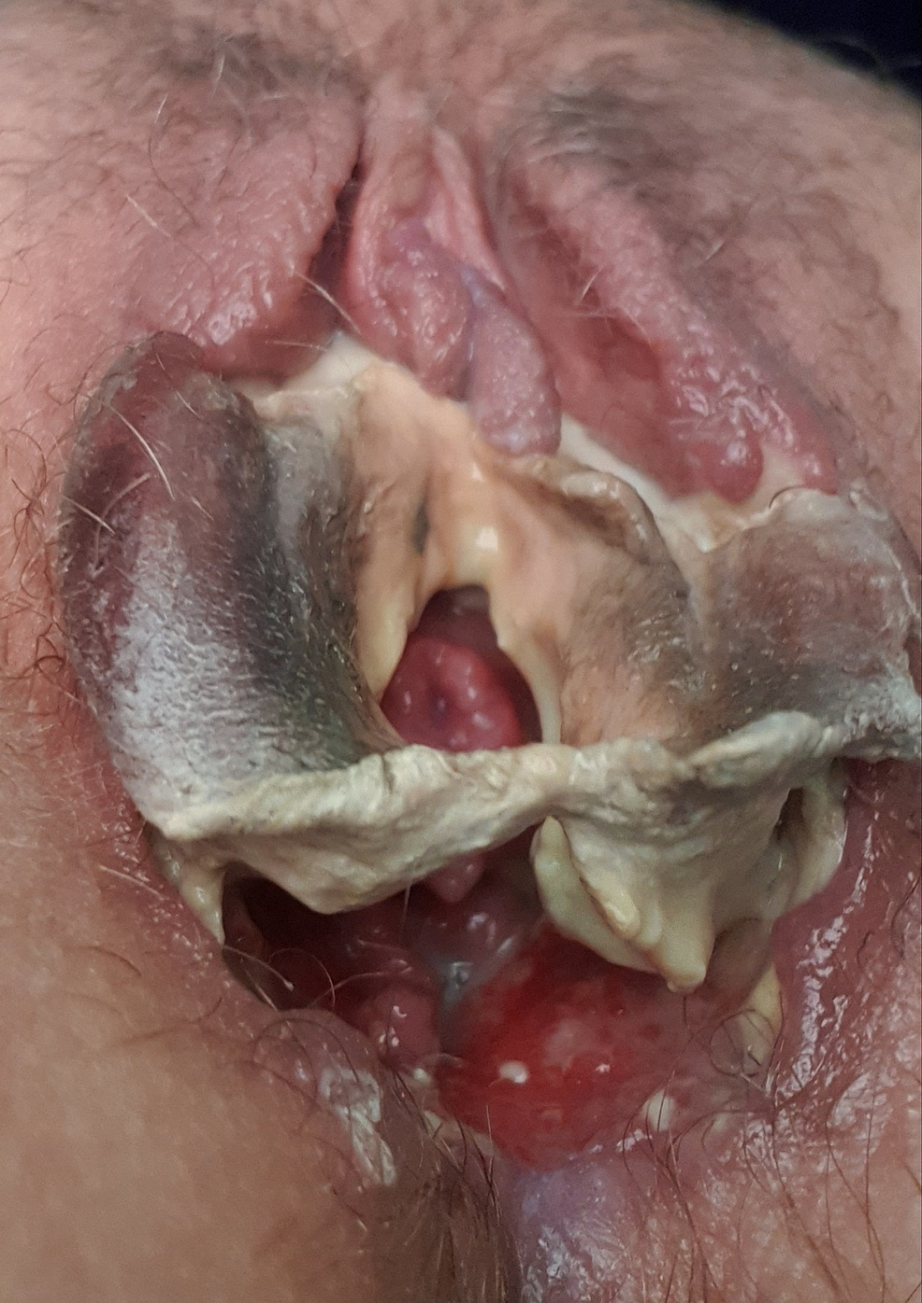
Female genitalia There are multiple deep ulcers with overlying yellow exudate and black eschar involving two-thirds of both labia majora and minora, sparing the clitoris and punched out unremarkable non violaceous borders.

A 31-year-old single woman, of Mediterranean descent from Lebanon, presented with deep, painful genital ulcers and multiple oral aphthae for two weeks. Her condition was accompanied by arthralgia of the knees and elbows, light flashes, and blurry vision in both eyes. Her past history was significant for recurrent genital lesions and photosensitivity. She had no history of sexual activity or recent travel. The review of systems was otherwise unremarkable. Examination showed well-defined punched-out erosions over the buccal and gingival mucosa and multiple deep ulcers with overlying yellow exudate and black eschar involving two-thirds of both labia majora and minora sparing the clitoris and punched-out unremarkable non-violaceous borders with bilateral inguinal lymphadenopathy (Figure 1).

**Figure 2:**
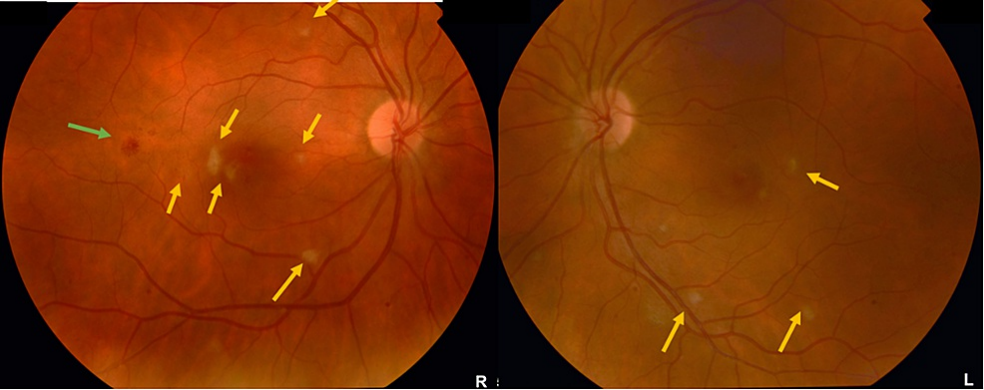
Color fundus photographs Color fundus photographs of the right and left eyes showing multiple white patches of retinal infarctions (yellow arrows) in addition to the intraretinal hemorrhage in the right eye (green arrow).

Pathergy test was negative. Ophthalmological examination showed reduced visual acuity in both eyes; 20/60 in the right eye and 20/30 in the left eye with quiet anterior segments. Dilated fundus examination showed few cotton wool spots and intraretinal hemorrhage. No evidence of vitritis, retinitis, or vasculitis was found (Figure 2).

**Figure 3:**
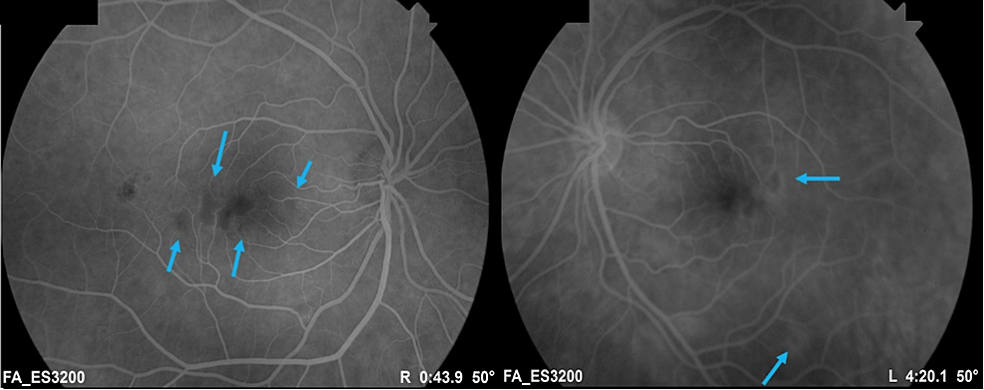
Fundus fluorescein angiography Fundus fluorescein angiography of the right and left eyes showing areas of capillary non-perfusion involving the macula in both eyes (blue arrows).

Fundus fluorescein angiography showed multiple arteriolar infarctions involving the macula in both eyes, more in the right eye. The retinal venous system did not appear to be affected (Figure 3).

**Figure 4:**
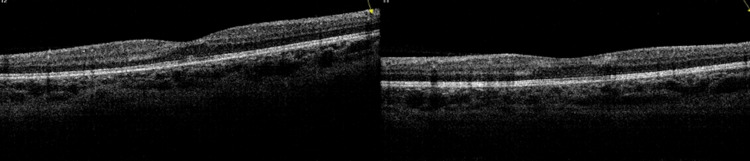
Optical coherence tomography Optical coherence tomography of the right (L) and left (R) eyes showing inner nuclear and outer plexiform layers hyper-reflectivity indicating ischemia.

Optical coherence tomography of the right and left eyes showing inner nuclear and outer plexiform layers hyper-reflectivity indicating ischemia (Figure 4).

Her immunological workup showed negative antinuclear antibody ANA, double-Stranded DNA, antineutrophil cytoplasmic antibodies ANCA, and complement C3 & C4. Tests for herpes simplex virus (HSV) IgM 1 and 2, hepatitis B and C viruses, cytomegalovirus (CMV) IgM, human immunodeficiency virus (HIV), QuantiFERON-TB, and syphilis were negative. The HLA B-51 was positive.

**Figure 5:**
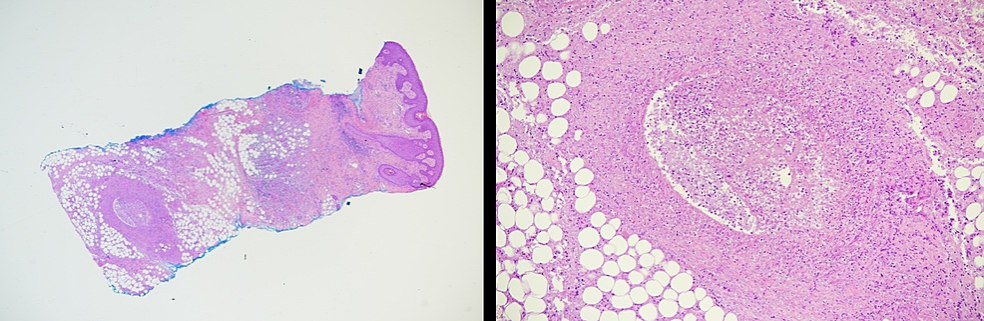
Deep skin biopsy Deep skin biopsy showed dermal and subcutaneous neutrophil infiltrate (left) (H and E, x20) and a medium-sized subcutaneous blood vessel with neutrophilic infiltration of its wall (H and E, x100) (right).

Microscopic examination of hematoxylin and eosin (H&E)-stained tissue sections from the vulvar biopsy revealed epidermal hyperplasia and dense dermal and subcutaneous inflammatory infiltrate with large numbers of neutrophils. A medium-sized subcutaneous artery was involved and showed neutrophilic infiltration of its wall. No granuloma was observed (Figure 5).

**Figure 6:**
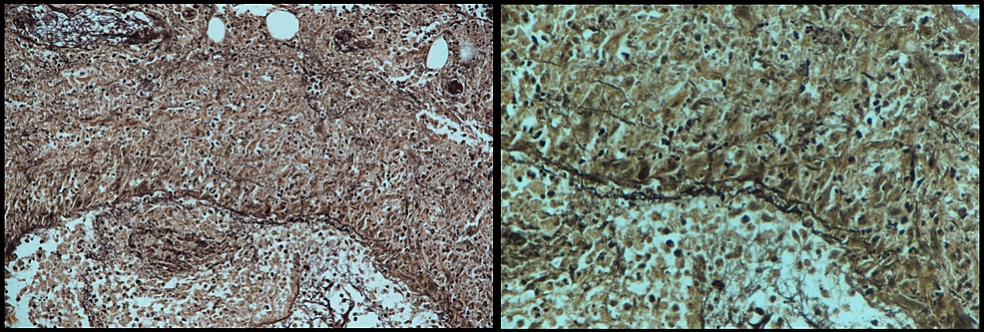
Stain for elastic lamina Stain for elastic lamina (left) showed medium-sized subcutaneous artery involvement (right).

Stain for elastic lamina showed medium-sized subcutaneous artery involvement (Figure 6).

**Figure 7:**
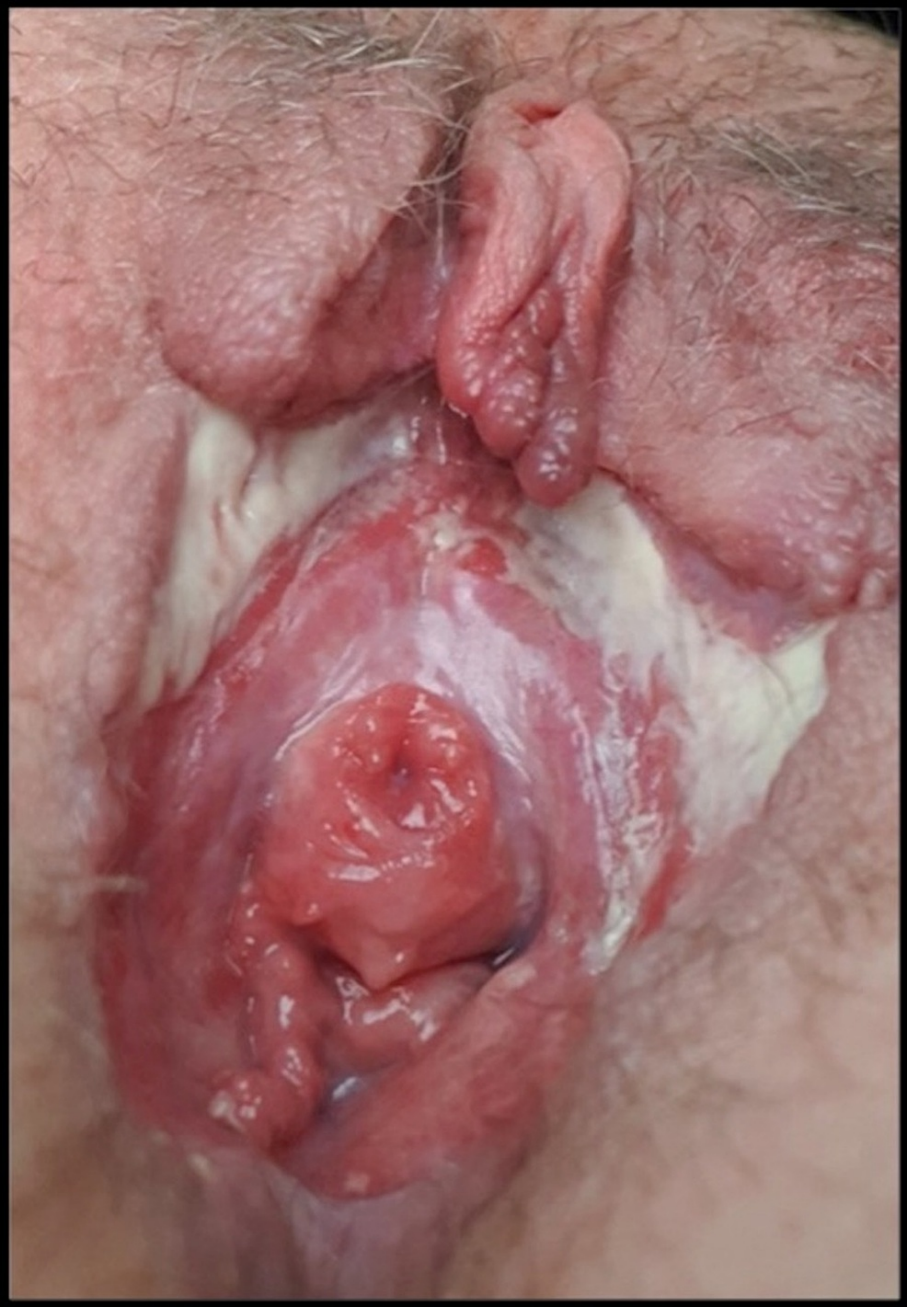
The ulcer Upon discharging the patient, the ulcer became shallower and the healing process started from the edges.

**Figure 8:**
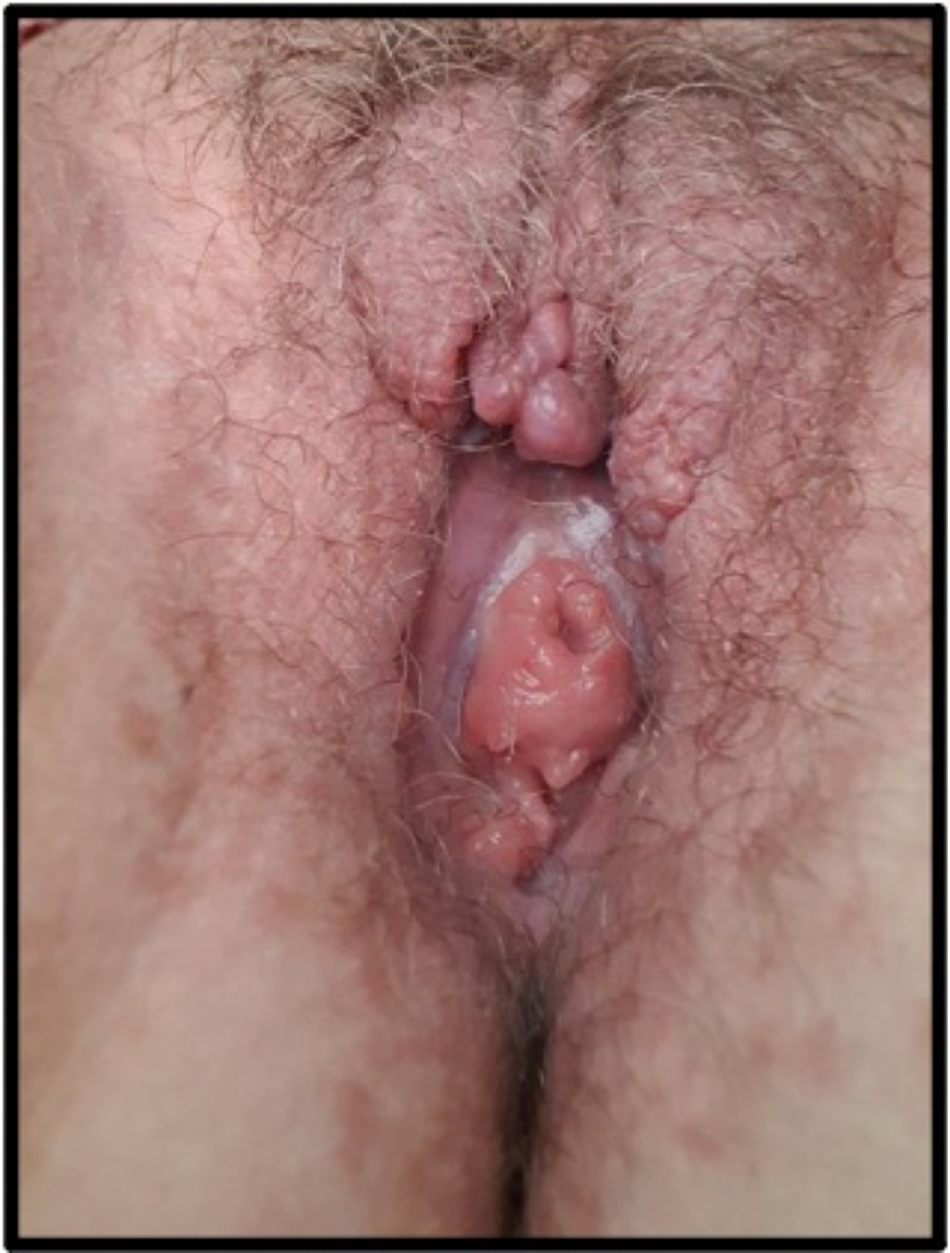
The ulcer Four months after being discharged from the hospital, there was marked improvement and healing of the ulcer, but distortion of the vulva.

Considering the above, a final diagnosis of the coexistence of polyarteritis nodosa in Behçet's disease patient was made. She was admitted and started on pain management, intravenous methylprednisolone 1,000 mg for three days suggested by the ophthalmologist to suppress the ocular damage and to stop further mutilation of the genitalia followed by oral prednisolone 50 mg (1 mg/kg) with a slow taper of 5 mg weekly, oral colchicine 0.5 mg twice daily, and adalimumab 40 mg once every two weeks. On follow-up after four months, there was sizeable improvement of the ulcer and no new cutaneous or systemic complaints reported (Figures 7-8). 

## Discussion

In most patients, PAN is idiopathic, however, in some, it can occur as a sequel of viral infections, mainly hepatitis B virus (HBV) [1]. Skin involvement in PAN usually manifests as tender erythematous nodules, purpura, livedo reticularis, ulcers, as well as bullous or vesicular eruptions [2].

Moreover, genital involvement can occur in PAN. In males, it manifests in the form of orchitis, with testicular tenderness in more than 10% of patients as well as chronic epididymitis, orchialgia, testicular hypersensitivity, or even necrotic ulcers in the foreskin and scrotum [12]. However, female genital involvement is described in the literature in about 110 cases only, usually affecting the internal genital organs; the cervix, uterus, and adnexa [13,3]. Indeed, only one case with vaginal ulceration was reported by Buekers et al. [4] Our patient’s ulcerative necrotic lesions, though not preceded by livedoid changes, had the histopathological findings of medium-sized artery involvement, making PAN a more likely diagnosis. Moreover, PAN ophthalmologic manifestations predominantly include scleritis, peripheral ulcerative keratitis, and retinal vasculitis. Other manifestations include non-granulomatous uveitis, pseudotumor of the orbit, and central retinal artery occlusion associated with temporal arteritis [14]. Ocular PAN can also present as acute bilateral visual loss [15]. It has been found that PAN, a disease with predominantly arterial involvement, affects the retinal arteries mostly, making them bear the brunt of the disease. Fundus fluorescein angiography of our patient showed multiple arteriolar infarctions involving the macula of both eyes while the retinal venous system was spared, in accordance with the findings of PAN. Moreover, the cotton wool spots that were noted in our patient’s examination reflect microinfarcts in the retina due to precapillary arteriolar occlusion as often found in association with systemic vasculitis such as SLE, granulomatosis with polyangiitis, Churg-Strauss syndrome, cryoglobulinemia, and PAN [16]. Therefore, we believe PAN is the underlying cause of her ocular manifestations. Interestingly, ocular involvement in PAN was found to affect prognosis and is indicative of more vigorous therapy regardless of other findings [17]. Histopathologic evidence of vascular inflammation in medium-sized or small arteries is crucial to the diagnosis of PAN. The inflammatory infiltrates are usually mixed and include neutrophils, lymphocytes, and macrophages [18]. Giant cells and granulomas are typically absent. In early and active lesions, neutrophils predominate and fibrinoid necrosis can be seen, while later stages usually show lymphocyte and macrophage infiltration [18]. This was demonstrated in our patient’s biopsy which showed medium-sized subcutaneous artery involvement with neutrophilic infiltration of its wall and no granulomas.

On the other hand, BD is a chronic relapsing autoimmune inflammatory disorder that involves different organ systems and causes recurrent oral and genital ulcers [5]. Genital ulcers usually involve the labia majora and minora in females, are painful, and tend to be larger and deeper, with more irregular borders and a longer healing duration than oral ulcers [19,20]. However, the genital lesions in our patient were very large and deep than usually described in the literature for BD. The involvement of a medium-sized subcutaneous artery is not in favor of BD as it involves veins more than arteries. However, when the arteries are involved in BD, it is more likely to be major arteries such as the aorta, pulmonary, femoral, iliac, coronary, and cerebral arteries [21]. Furthermore, ocular BD presents as diffuse capillary and venous inflammation with areas of retinal necrosis and major vascular occlusion [16,22]. The absence of anterior uveitis, vitritis, and retinal vasculitis in our patient, which would be common findings in BD, make it less likely as the cause of her findings [6].

PG presents as a deep ulcer with a well-defined undermined border, usually violet with surrounding erythematous skin [23]. The clinical evolution is characteristic; it starts as a small papule or collection of papules, which break down to form small ulcers [23]. These findings were not seen in our patient. While ocular PG is rarely reported in the literature, it can present most commonly with peripheral ulcerative keratitis [24], which was also not seen in our patient.

Few reports have demonstrated the coexistence of cutaneous PAN in BD patients [11]. Heller described a BD patient with subcutaneous nodules on the lower legs histologically consistent with cutaneous PAN [8]. While Vikas et al. reported a BD patient with recurrent cutaneous polyarteritis-nodosa-like lesions and suggested that cutaneous PAN could be a marker of severe BD [9]. Moreover, Serra-Guillén et al. reported the onset of cutaneous PAN-like in their patient with BD as a marker of worsening disease [10].

The treatment of PAN is directed based on the subtype and the severity of the disease. The main medication used for the treatment of PAN is a glucocorticoid. In some cases, cyclophosphamide was used along with glucocorticoid; the remission rate on that regimen reached up to 90% [25]. With regards to cutaneous PAN, glucocorticoid again was considered the first-line treatment, especially for painful or ulcerative lesions. The high-quality studies on steroid-sparing agents are lacking, yet reviewed data from several case reports showed potential to great efficacy of some conventional DMARDs (methotrexate, leflunomide, hydroxychloroquine, colchicine, and azathioprine) [26]. Furthermore, the use of other biologics like anti-TNF, rituximab, and tofacitinib and have been reported in the literature with varying reported responses [27,28]. A recent systematic review published in 2020 showed that tocilizumab can be an effective treatment for refractory cases or for patients with severe manifestations [29].

Given our patient’s presentation of disfiguring genital ulcers and ocular involvement, we decided to treat her aggressively with high-dose steroids. Enough evidence in the literature suggests that PAN appears to be responsive to anti-TNF therapy, given the high TNF levels in affected patients [30]. In fact, several reports showed the efficacy of adalimumab in treating both refractory and cutaneous PAN [31, 32]. The addition of adalimumab to our patient appeared to arrest the disease progression and severity.

## Conclusions

To conclude, this is a report of a case of atypical PAN coexisting with BD. Our differential, in this case, was pyoderma gangrenosum and severe vulval necrosis as part of BD and PAN. The presence of cutaneous medium-size arteries' involvement, the presence of occlusive vasculopathy of the retina, and the lack of the typical retinal findings of BD favored the diagnosis of PAN as part of the cutaneous manifestations of BD. Therefore, we agree with other published literature that PAN could be one of the manifestations of BD. By reporting this case, we aim to stress the need to treat vigorously when PAN presents itself with such a clinical picture to avoid serious complications. Moreover, to our knowledge, there is yet no literature report that describes vulvar PAN, which makes this case unique.
